# Model selection in Medical Research: A simulation study comparing Bayesian Model Averaging and Stepwise Regression

**DOI:** 10.1186/1471-2288-10-108

**Published:** 2010-12-06

**Authors:** Anna Genell, Szilard Nemes, Gunnar Steineck, Paul W Dickman

**Affiliations:** 1 Clinical Cancer Epidemiology, Department of Oncology, Institute of Clinical Sciences, Sahlgrenska University Hospital, Gothenburg, Sweden; 2 Clinical Cancer Epidemiology, Karolinska Institutet, Karolinska University Hospital, Stockholm, Sweden; 3 Department of Medical Epidemiology and Biostatistics, Karolinska Institutet, Stockholm, Sweden

## Abstract

**Background:**

Automatic variable selection methods are usually discouraged in medical research although we believe they might be valuable for studies where subject matter knowledge is limited. Bayesian model averaging may be useful for model selection but only limited attempts to compare it to stepwise regression have been published. We therefore performed a simulation study to compare stepwise regression with Bayesian model averaging.

**Methods:**

We simulated data corresponding to five different data generating processes and thirty different values of the effect size (the parameter estimate divided by its standard error). Each data generating process contained twenty explanatory variables in total and had between zero and two true predictors. Three data generating processes were built of uncorrelated predictor variables while two had a mixture of correlated and uncorrelated variables. We fitted linear regression models to the simulated data. We used Bayesian model averaging and stepwise regression respectively as model selection procedures and compared the estimated selection probabilities.

**Results:**

The estimated probability of not selecting a redundant variable was between 0.99 and 1 for Bayesian model averaging while approximately 0.95 for stepwise regression when the redundant variable was not correlated with a true predictor. These probabilities did not depend on the effect size of the true predictor. In the case of correlation between a redundant variable and a true predictor, the probability of not selecting a redundant variable was 0.95 to 1 for Bayesian model averaging while for stepwise regression it was between 0.7 and 0.9, depending on the effect size of the true predictor. The probability of selecting a true predictor increased as the effect size of the true predictor increased and leveled out at between 0.9 and 1 for stepwise regression, while it leveled out at 1 for Bayesian model averaging.

**Conclusions:**

Our simulation study showed that under the given conditions, Bayesian model averaging had a higher probability of not selecting a redundant variable than stepwise regression and had a similar probability of selecting a true predictor. Medical researchers building regression models with limited subject matter knowledge could thus benefit from using Bayesian model averaging.

## Background

Automatic variable selection methods are usually discouraged in medical research although we believe they might be valuable for studies where subject matter knowledge is limited. Bayesian model averaging [1] may be useful for model selection; it may be worthwhile to further investigate its performance compared to commonly used automatic selection procedures such as stepwise regression.

The context of this study is a class of observational studies where we hope to identify predictors of a single outcome from within a range of 20-40 possible explanatory variables. We are particularly interested in the situation where we have been the first, or among the first, to collect empirical data in a research field and subject matter knowledge is therefore nonexistent or extremely limited. Our interest is not on testing a limited number of well-defined hypotheses but on describing associations between potential predictors and the outcome. It is, if not impossible, hard to manually assess all combinations of predictor variables even when we ignore the possibility of interactions. In such scenarios there are strong arguments for making use of data-driven model selection methods (ideally in conjunction with subject matter knowledge if there is any). In this context false positives (type I error) can be a major problem [2-4].

The most well-known and widely-applied such method is stepwise regression which has been shown to perform poorly in theory, case-studies, and simulation [2-6]. Also, it is generally desirable to validate each step of the model building process [7] including model selection.

Wang and coworkers compared, in a simulation study [8], Bayesian model averaging to stepwise regression. They found that Bayesian model averaging 'chose the optimal model eight to nine out of ten simulations'. However, they did not perform more than ten simulations, so the possibility that their conclusions were dependent on random chance cannot be excluded. Also, Wang and coworkers did not mention any controlling or variation of the effect size of a true predictor and they did not examine the situation where a redundant variable is correlated with a true predictor. Also Raftery and coworkers performed a simulation study [2] and found that in ten simulations of a null model (no predictor variables were related to the outcome variable), the built-in selection method ("Occam's window") in Bayesian model averaging chose the null model or models with just a few variables whereas stepwise regression chose models with many variables.

The classical *α *-level of 0.05 is historically accepted and is a convention in the scientific community. One might intuitively use the posterior probability in BMA in a similar way and therefore use a 95% threshold although the convention in the Bayesian model averaging litterature is using a 50% posterior probability threshold as analogous to the frequentist 0.05 significance level [9,10].

In this study we use linear regression to examine and compare stepwise regression (using Akaike Information Criterion (AIC) for model building together with 0.05 significance criteria for inclusion in the final model) with Bayesian model averaging (applying both a 50% and a 95% posterior probability threshold) in terms of selecting true predictors and redundant variables by simulating data corresponding to five different data generating processes and thirty different values of effect size of a true predictor and then analyzing the simulated data with Bayesian model averaging and stepwise regression respectively. We chose to perform our study in the framework of linear regression to facilitate greater control of the effect size of true predictors (the parameter estimate divided by its standard error).

## Methods

### Data simulation

We designed 5 different data generating processes that can be said to represent a hypothetical cross-sectional study containing one outcome, Y, which was conditioned on 0, 1 or 2 of the 20 remaining variables *X*_1_, ... *X*_20_.

**Figure 1:**
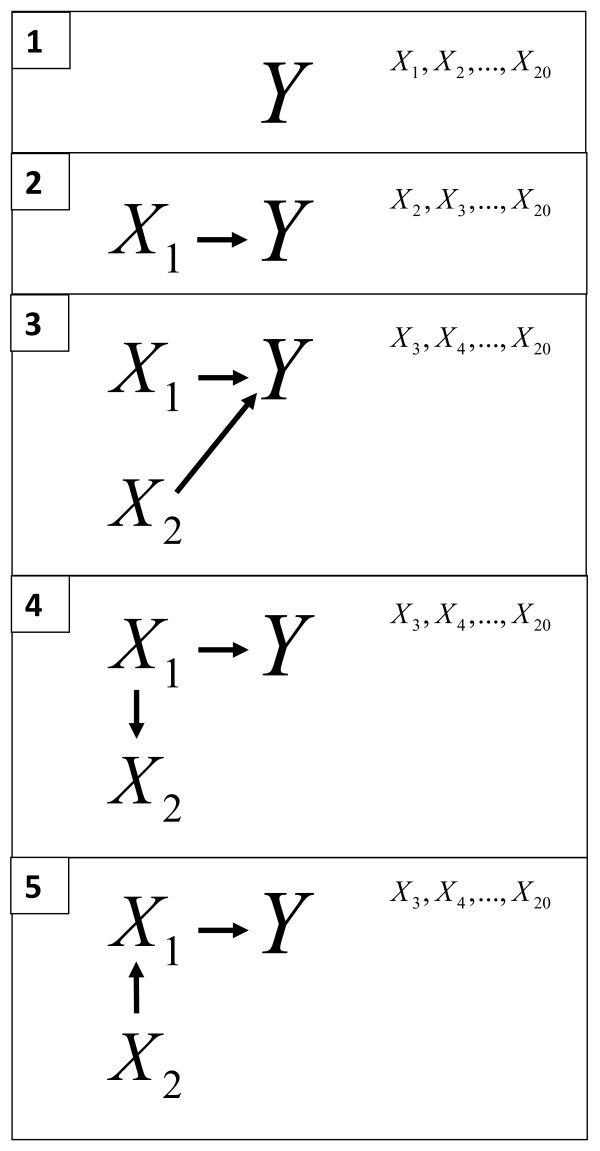
**Graphical view over data generating processes**. Graphical presentation of the data generating processes 1, 2, 3, 4 and 5.

The outcome *Y_jkl _*was generated as follows: $Y_{jkl}=\sum_{i=1}^{20}\beta_{i}x_{ijkl}+\varepsilon_{jkl}$, where *i *denotes twenty different variables, *j *denotes five data generating processes, *k *denotes thirty different values of the residual variance, *l *denotes 300 simulations and *ε_jkl _~ N*(0, *σ_k_*), *σ*_1 _= 0.5,...,*σ*_30 _= 80 with the increment 2.74. The five data generating processes, which are described graphically in Figure 1 were specified as

1. *β_i _*= 0 ∀*i*

2. *β*_1 _= 1 and *β_i _*= 0 ∀*i *> 1

3. *β*_1 _= 1, *β*_2 _= 1 and *β_i _*= 0 ∀*i *> 2

4. *β*_1 _= 1 and *β_i _*= 0 ∀*i *> 1 and $X_{2}=X_{1jkl}+\varepsilon_{X_{2jkl}}$, where $\varepsilon_{X_{2jkl}}$ ~ *N*(0, 1)

5. *β*_1 _= 1 and *β_i _*= 0 ∀*i *> 1 and $X_{1}=X_{2jkl}+\varepsilon_{X_{1jkl}}$, where $\varepsilon_{X_{1jkl}}$ ~ *N*(0, 1)

In each of a series of 300 simulations we commenced by generating 500 observations of 20 independent, identically distributed random variables from a standard normal distribution. For data generating process 4, the redundant variable *x*_2 _was generated from the true predictor *x*_1_, and in data generating process 5 the true predictor *x*_1 _was generated from *x*_2_. Therefore, *x*_2 _in data generating process 4 and *x*_1 _in data generating process 5 did not have standard normal distributions.

We define variables used for generating the outcome as true predictors and the remaining variables as redundant variables. We varied the effect size of the true predictor (where we define effect size as the parameter estimate divided by its standard error) by adding to the data generating process an error variable with mean zero and a range of 30 different values of the variance. We regard the data generating process 1 as being less complex than data generating process 2, which in turn is less complex than data generating process 3, and so on.

We repeated the simulation independently (i.e., simulated new values of *x*_1_, ... *x*_20_) from each data generating process all 300 times for the 30 different values of sigma. We varied the effect size by varying sigma because for a fixed *β *, the effect size of the true predictor (and thus the probability of selecting a true predictor) is dependent on the amount of noise. The simulations were performed in R [11] using the function rnorm (which uses the Mersenne-Twister random number generator). A random seed was generated for each simulation.

### Data analysis

For each of the five data generating processes and each of the 30 values of effect size, we analyzed each of the 300 simulated data sets using both Bayesian model averaging and forward stepwise regression selection with the Akaike Information Criterion (AIC) as the step criteria [12]. As a final step in stepwise regression we excluded all previously selected variables with a p-value of 0.05 or greater. We will refer to this as stepwise regression. In Bayesian model averaging we made use of the posterior probabilities given for each variable and introduced on one hand a 95% threshold and on the other hand a 50% threshold for the posterior probabilities for the variables in the averaged model. The 95% threshold was motivated by the approach a first time user might naively take. The 50% threshold was motivated by convention in the Bayesian model averaging literature [9,10]. We thus defined variables having a posterior probability below 95% and 50% respectively as selected. We aimed to study a situation where subject matter knowledge is extremely limited. When analysing the data we assumed no existing subject matter knowledge. Therefore, for Bayesian model averaging we used noninformative priors. Further, we used Gaussian error distribution, constant equal to 20 in the first principle of Occam's window and non-strict Occam's window. The analyses were performed in R [11] using the lm and step functions and, for Bayesian model averaging, the bic.glm function in the BMA package [13,14]. An overview of Bayesian model averaging is given in the appendix.

### Method comparison

We compared the selection methods in terms of the probability of selecting a true predictor and the probability of not selecting a redundant variable. For each method, we estimated the probability of selecting a true predictor as the proportion of cases where a true predictor was selected and the probability of not selecting a redundant variable as the proportion of cases where a redundant variable was not selected. We also compared the probability of selecting the correct model which we estimated as the proportion of cases where the true predictor or predictors (in the case of two true predictors) was selected and no other variables were selected. For the null hypothesis *H*_0 _: *β_i _*= 0, the probability of selecting a true predictor corresponds to the probability of rejecting *H*_0 _given it is false and the probability of not selecting a redundant variable corresponds to the probability of failing to reject *H*_0 _given it is true.

## Results

### Probability of not selecting a redundant variable

**Figure 2:**
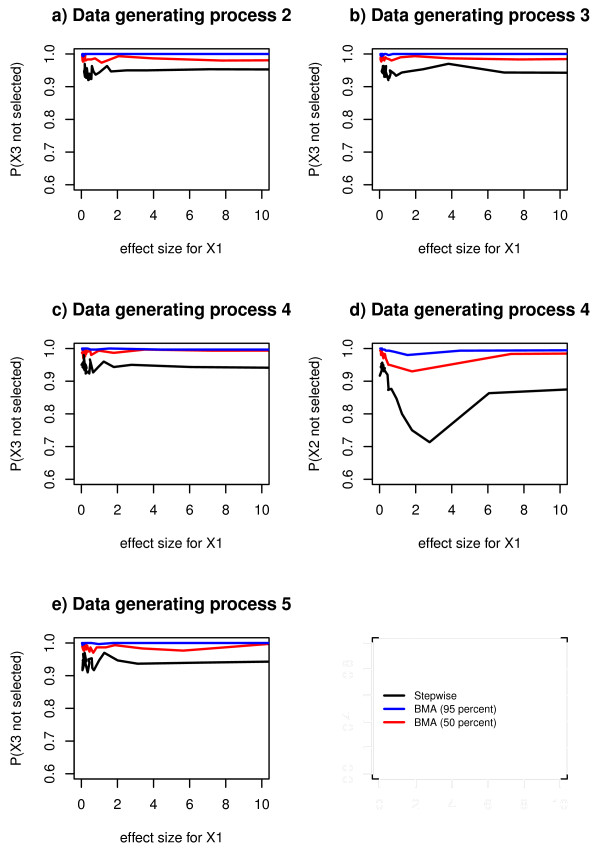
**Estimated probabilities of not selecting a redundant variable, for comparison between selection methods**. Estimated probabilities of not selecting a redundant variable in Bayesian model averaging with 95% threshold, Bayesian model averaging with 50% posterior probability threshold and stepwise regression, for 30 different values of the effect size, in data generating process 2, 3, 4 and 5.

For data generating process 1, Bayesian model averaging with 95% threshold almost never (less than 1 time per hundred) selects redundant variables, Bayesian model averaging with 50% threshold selects a redundant variable 1 time per hundred and stepwise regression selects a redundant variable with probability 0.05 (data not shown). These probabilities are independent of the effect size of the true predictor. This holds for the other data generating processes when considering redundant variables that are uncorrelated with a true predictor (Figure 2a, b, c and 2e). The exception to the pattern was data generating process 4 when a redundant variable was correlated with a true predictor. In this case the probability of selecting a redundant variable was dependent on the effect size of the true predictor. Above an effect size corresponding to a t-test statistic of 2, the probability of not selecting a redundant variable varied between approximately 0.7 and 0.9 for stepwise regression. For Bayesian model averaging with 50% threshold it varied between approximately 0.8 and 1. For Bayesian model averaging with 95% threshold it was approximately 1(Figure 2d).

### Probability of selecting a true predictor

**Figure 3:**
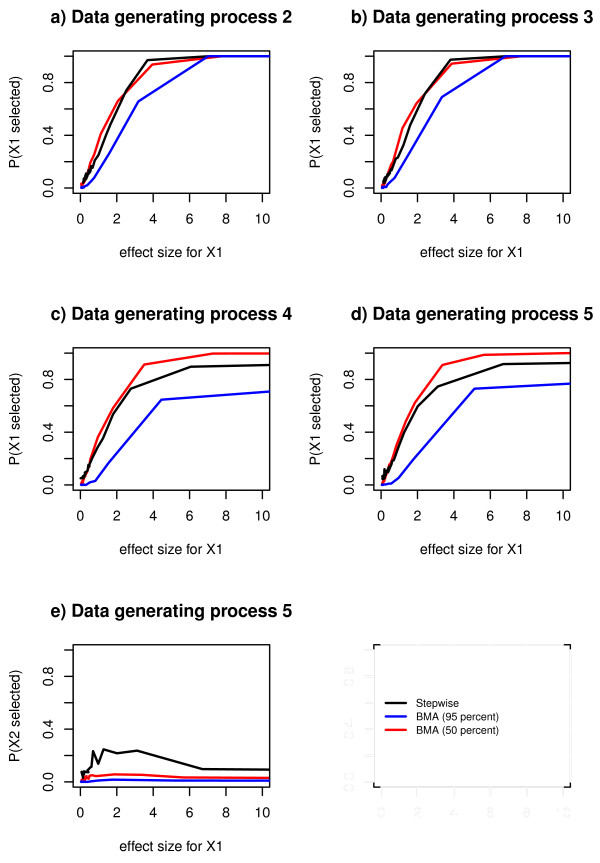
**Estimated probabilities of selecting a true predictor, for comparison between selection methods**. Estimated probabilities of selecting a true predictor in Bayesian model averaging with 95% threshold, Bayesian model averaging with 50% posterior probability threshold and stepwise regression, for 30 different values of the effect size, in data generating process 2, 3, 4 and 5.

We observed that the probability of selecting a true predictor increased as the effect size of the true predictor increased (Figure 3). For data generating processes 2 and 3, Bayesian model averaging with 50% threshold and Stepwise regression performed similarly and better than Bayesian model averaging with 95% threshold (Figure 3a-d). For the data generating processes 4 and 5 Bayesian model averaging with 50% threshold performed best, followed by Stepwise regression (Figure 3c and 3d).

For data generating processes 2 and 3, the probability of selecting a true predictor leveled out at 1 for both stepwise regression and Bayesian model averaging (both with 95% threshold and with 50%) (Figure 3a and 3b). For the data generating processes 4 and 5 this selection probability also leveled out at 1 for Bayesian model averaging with 50% threshold. For stepwise regression it leveled out at 0.9. For Bayesian model averaging with 95% threshold it leveled out at approximately 0.7 (Figure 3c and 3d).

### Probability of selecting an indirect predictor (Data generating process 5)

For Bayesian model averaging with 95% threshold the probability of selecting an indirect predictor (*x*_2 _in data generating process 5) was approximately constant at 0 but for stepwise regression it increased to approximately 0.2 for effect size corresponding to a t-test statistic between 0 and 3 and at t-test statistic of approximately 7 the probability decreased and leveled out at approximately 0.1 (Figure 3e). For Bayesian model averaging with 50% threshold this probability varied between 0.01 and 0.06 (Figure 3e).

### Probability of selecting correct model

**Figure 4:**
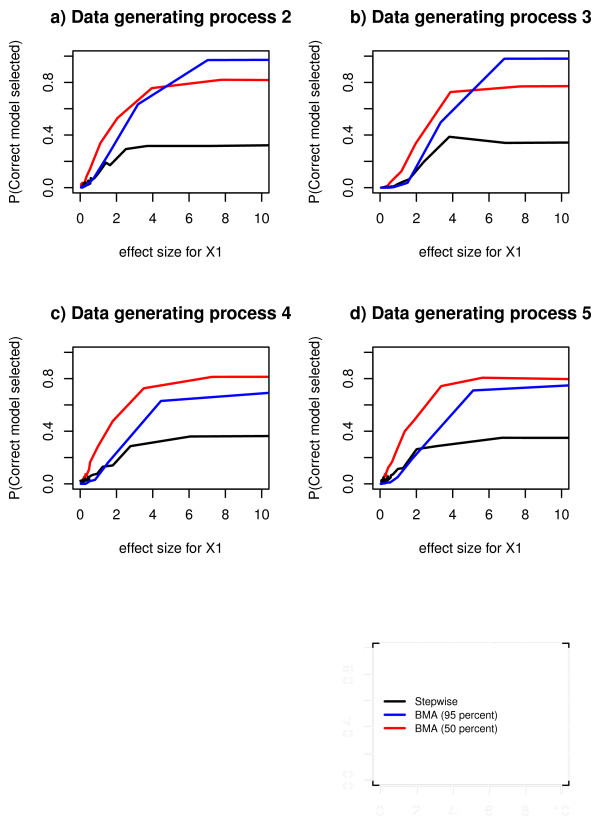
**Estimated probabilities of selecting correct model, for comparison between selection methods**. Estimated probabilities of selecting correct model in Bayesian model averaging with 95% threshold, Bayesian model averaging with 50% posterior probability threshold and stepwise regression, for 30 different values of the effect size, in data generating process 2, 3, 4 and 5.

The probability of selecting the correct model increased as the effect size of the true predictor increased but the methods differed (Figure 4). Stepwise regression generally leveled out at selection probability approximately 0.3 in all data generating processes (Figure 4a-d). Bayesian model averaging with 95% threshold leveled out at approximately 1 for data generating processes 2 and 3 (Figure 4a and 4b) and on probability between 0.7 and 0.8 for data generating processes 4 and 5 (Figure 3c and 3d). Bayesian model averaging with 50% threshold leveled out at approximately 0.8 in all data generating processes (Figure 4a-d).

### Influence of model complexity on selection probabilities

**Figure 5:**
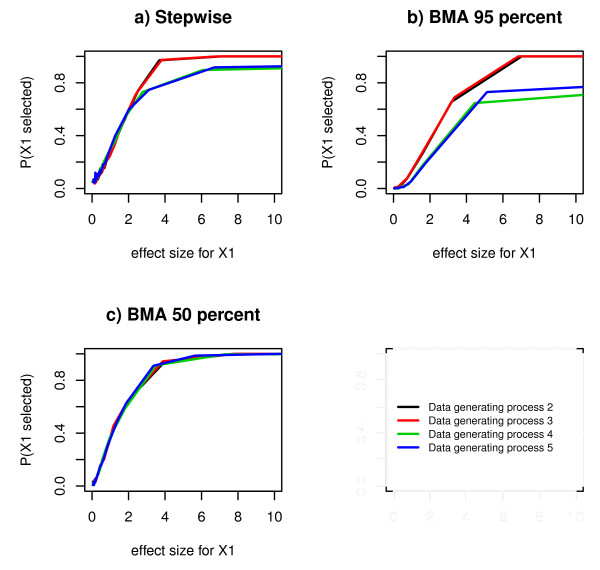
**Estimated probabilities of selecting a true predictor, for comparison within selection methods, between model complexities**. Estimated probabilities of selecting a true predictor in data generating process 2, 3, 4 and 5 for 30 different values of the effect size, in Bayesian model averaging with 95% threshold, Bayesian model averaging with 50% posterior probability threshold and stepwise regression.

For data generating processes 4 and 5, the probability of selecting a true predictor in stepwise regression leveled out at a probability of approximately 0.9 compared to a probability of approximately 1 for data generating processes 2 and 3 (Figure 5a). In Bayesian model averaging with 95% threshold the probability of selecting a true predictor leveled out at a probability of approximately between 0.6 and 0.7 for data generating processes 4 and 5 compared to a probability of approximately of approximately 1 for data generating processes 2 and 3 (Figure 5b). In Bayesian model averaging with 50% threshold the probability of selecting a true predictor leveled out at 1 for all data generating processes (Figure 5c).

## Discussion

We simulated data from five different pre-determined data generating processes, for 30 different values of the effect size (the parameter estimate divided by its standard error), and analyzed the simulated data with stepwise regression (using Akaike Information Criterion (AIC) for model building together with 0.05 significance criteria for inclusion in the final model) and Bayesian model averaging (applying both a 50% and a 95% posterior probability threshold) respectively. We found that Bayesian model averaging almost never selected a redundant variable, whereas stepwise regression did - 1 time out of 20 for a redundant variable not correlated with the true predictor. Even a redundant variable which correlates with the true predictor was less often selected by Bayesian model averaging than by stepwise regression which sometimes selected such a variable more than 1 time out of 4, depending on effect size. Bayesian model averaging with 50% posterior probability threshold performed similar to stepwise regression in selecting a true predictor. The probability of selecting a true predictor depended on effect size of the true predictor. Bayesian model averaging almost never selected an indirect predictor, while on the contrary stepwise regression did, depending on effect size. We noted that Bayesian model averaging with 95% posterior probability threshold is less likely to select a true predictor than Bayesian model averaging with 50% posterior probability threshold. Since the convention in the Bayesian model averaging literature is to use 50% posterior probability as a threshold, we focus discussion on comparison between stepwise regression and Bayesian model averaging with 50% posterior probability threshold.

Our notion that Bayesian model averaging is less likely than stepwise regression to select redundant variables is consistent with two previously published simulation studies. In a study [8] with 10 simulations corresponding to our data generating processes 1 and 3, Wang and colleagues found Bayesian model averaging was less likely to select redundant variables than stepwise regression backward elimination. Despite differences in the study designs (logistic vs. linear regression and fewer simulations) their results are consistent with ours in supporting the notion that Bayesian model averaging is less likely to select redundant variables than stepwise regression. Viallefont and coworkers performed a similar study [15], based on 200 simulations, providing further support. They simulated data from a data generating process similar to our number 4 except with 50 variables of which 10 were true predictions and some correlated with each other. Making use of stepwise regression backward elimination, they fitted logistic regression models and reported their results in terms of the proportion of selected variables that were true predictors. They found stepwise regression more likely to select redundant variables than Bayesian model averaging. Of the variables selected (in the p-value intervals < 0.001, 0.001-0.01 and 0.01-0.05) by stepwise regression, 86% of them were true predictors whereas 98% were true among those selected by Bayesian model averaging (in the posterior probability intervals 95-99% and > 99%). Since those variables selected by Bayesian model averaging contained a lower proportion of false positives, we can conclude that the probability of selecting a redundant variable was lower for Bayesian model averaging than for stepwise regression. A study by Raftery and coworkers [2] also supports this conclusion. They simulated 50 standard normal redundant variables not related to a standard normal outcome variable, repeated the simulation 10 times and found that "In five simulations, Occam's window chose only the null model. For the remaining simulations, three models or fewer were chosen along with the null model". On the other hand "stepwise method chose models with many predictors". In an earlier paper [13] Raftery and coworkers made a similar experiment which gave a similar result. In our study, we note that stepwise regression selected a redundant variable which correlates with a true predictor more often than it selected an uncorrelated variable, whereas Bayesian model averaging did not select a redundant variable even if it was correlated with a true predictor. The studies mentioned above [2,8,15] did not present probabilities of selecting a correlated redundant variable but Bayesian Model averaging is known to favor smaller models [16] and this supports our notion that Bayesian Model averaging is less likely than stepwise regression to select a redundant variable, be it uncorrelated or correlated with a true predictor.

The study by Wang and coworkers [8] also compared the probabilities of selecting a true predictor. In that work, both Bayesian model averaging and stepwise regression selected the two true predictors 10 out of 10 times. The study by Raftery and coworkers [2] provide some further support for the finding that Bayesian model averaging has a similar probability of selecting a true predictor as stepwise regression. Raftery and coworkers simulated a data set with one true predictor and 29 redundant variables. Occam's window chose the correct model. Stepwise regression chose a model with two variables - the true predictor together with a redundant variable. In the earlier study [13] Raftery and coworkers made two simulations of an outcome variable dependent on one true predictor but not related to 49 redundant variables. Both stepwise regression and Occam's window selected the true predictor. Our study, with 300 simulations, shows that Bayesian model averaging with a posterior probability threshold of 50% has a similar probability of selecting a true predictor as stepwise regression. Available data thus show that the higher probability of not selecting a redundant variable in Bayesian model averaging compared to stepwise regression does not come at the price of lower probability of selecting a true predictor but instead provides us with similar probability of selecting a true predictor as stepwise regression. Bayesian model averaging almost never selected an indirect predictor, whereas stepwise regression did, depending on effect size. Neither the Wang study [8] nor the study [15] by Viallefont presented probabilities of selecting an indirect predictor. The difference between stepwise regression and Bayesian model averaging in selecting an indirect predictor was similar to the difference between the methods in the case with selection of the correlated redundant variable. This is not surprising since the two phenomena (selecting a correlated redundant variable and selecting an indirect predictor) are mathematically similar. Yamashita and colleagues [12] have given theoretical arguments and recommend that two highly correlated variables should not be entered into a selection procedure at the same time. Ideally, one of them should, based on subject matter knowledge, be chosen.

The probability of selecting the correct model was substantially lower for stepwise regression than for Bayesian model averaging. The Wang study [8] reported that Bayesian model averaging selected the correct model 9 out of 10 times whereas stepwise regression only did 3 times out of 10. We do not, in our study, see a clear picture when comparing the probabilities of selecting the correct model in Bayesian model averaging with 95% and Bayesian model averaging with 50%. However, based on available information we can conclude that Bayesian model averaging performs better than stepwise regression in selecting the correct model.

While Bayesian model averaging was primarily developed as a method for model averaging and handling model uncertainty, we chose to explore the use of Bayesian model averaging as a model selection method. Kass and Raftery [9] offer informative thresholds for interpreting posterior probabilities, providing us with the convention that the posterior probability threshold 50% corresponds to the 0.05 p-value significance level. In our simulation study we use linear regression because it allowed us to directly control the variance independently of the regression coefficient and thus to control the effect size. We regard this as a strength of this study since none of the previously published studies comparing Bayesian model averaging and stepwise regression presented results for different values of the effect size. With our simulations we tried to mirror simple data generating processes that can be said to be basic components of what one encounters in medical research. We deliberately chose data generating processes that were small and simple in order to more easily see differences between the model selection methods. Also, using a real life data set would not allow variation of the effect size. We view our chosen data generating processes as the basic building blocks of what one encounters in real life, although recognize that the findings from our simple scenarios may not translate perfectly to real life. There is also a need for a more nuanced covariance structure. In our study, the probabilities of not selecting a redundant variable, not correlated with a true predictor, in both stepwise regression and Bayesian model averaging are approximately constant over changes in complexity of the data generating process. For stepwise regression, however, the probabilities of selecting a true predictor are higher for lower complexity of the data generating process and gradually decrease with increasing complexity, whereas the probabilities of selecting a true predictor in Bayesian model averaging with 50% posterior probability threshold does not show this sensitivity to increasing complexity. If this advantage of Bayesian model averaging should persist even in the most complex real life data structures, it would add to the evidence in favor of Bayesian model averaging. This aspect deserves more attention and could be the topic of a future study of these methods.

## Conclusion

Our simulation study showed that under the given conditions, Bayesian model averaging had a higher probability of not selecting a redundant variable than stepwise regression and had a similar probability of selecting a true predictor. Medical researchers building regression models with limited subject matter knowledge could thus benefit from using Bayesian model averaging.

## Competing interests

The authors declare that they have no competing interests.

## Authors' contributions

AG and PD conceived the study. AG participated in its design, carried out its implementing and drafted the first version of the manuscript. PD participated in study design. SzN participated in study implementation. GS and PD coordinated the study. All authors contributed to the writing and approved the final version.

## Appendix

### Bayesian model averaging

As described by Hoeting and coworkers [1], instead of basing inference on one single model, Bayesian model averaging takes into account all the models considered. For some quantity of interest *θ*, such as a regression coefficient, the inference about *θ *is not only based on one single selected model but on the average of all possible models. For the quantity of interest *θ *the posterior distribution given data D is

(1) $$p(\theta |D)=\sum_{k=1}^{K}p(\theta |M_{k},D)p(M_{k}|D)$$

This is an average of the posterior distributions under each of the *K *models considered - a sum of terms where each term is the posterior *θ*-probability given data *D *and a model *M_k_*, weighted by the probability for that model *M_k _*given data *D*.

So inference about *θ *is not only based on one single selected model but on an average of posterior distributions of all identified models. In this way Bayesian model averaging accounts for model uncertainty. In (1) the posterior probability for model *M_k _*is given by

(2) $$p(M_{k}|D)=\frac{p(D|M_{k})p(M_{k})}{\sum_{l=1}^{K}p(D|M_{l})p(M_{l})}$$

where

(3) $$p(D|M_{k})=\int p(D|\xi_{k},\;M_{k})p(\xi_{k}|M_{k})d\xi_{k}$$

is the integrated likelihood of model *M_k_*,*ξ_k _*is the vector of parameters of model *M_k_*, *p*(*ξ_k_*|*M_k_*) is the prior density of *ξ_k _*under model *M_k_*, *p*(*D*|*ξ_k_*), *M_k _*is the likelihood and *p*(*M_k_*) is the prior probability that *M_k _*is the true model.

The sum in (1) can be exhaustive. An approach for managing the summation is to average over a subset of models that are supported by data. One method for this is called the Occam's window [17].

This is a method of accepting the models which are most likely to be the true model and not accepting any unnecessarily complicated model. Two principles that form the basis for the method are briefly presented here.

1. When comparing two models, the one that predicts data far less well than the better model should no longer be considered. A more formal way of saying this is that models not belonging to the set *S*1, where

(4) $$S1=\{M_{k}:\frac{\max_{l}\{p(M_{l}|D)\}}{p(M_{k}|D)}\leq C\}$$

should be excluded from (1). *C *is chosen by the data analyst.

2. If a model is simpler or smaller than a model it is being compared with and data provides evidence for the simpler model, then the more complex model should no longer be considered. Thus models should also be excluded if they belong to the set *S*2, where

(5) $$S2=\{M_{k}:\exists M_{l}\in S1,\;M_{l}\subset M_{k},\frac{p(M_{l}|D)}{p(M_{k}|D)}>1\}$$

Then (1) is replaced by

(6) $$p(\theta |D)=\sum_{{}_{M_{k}\in S}}p(\theta |M_{k},D)p(M_{k}|D),$$

where *S *= *S*1\*S*2 and all probabilities will implicitly be conditional on the the set of models in *S*. The consensus now in the Bayesian model averaging literature is not to use the second principle.

The BMA package in R [11] is an implementation of the Bayesian model averaging method.

## Pre-publication history

The pre-publication history for this paper can be accessed here:

http://www.biomedcentral.com/1471-2288/10/108/prepub
